# Supplementary material for: The adaptive benefit of evolved increases in hemoglobin-O2 affinity is contingent on tissue O2 diffusing capacity in high-altitude deer mice
Source: BMC Biol. 2021 Jun 22;19:128. doi: 10.1186/s12915-021-01059-4 (PMC8218429; doi:10.1186/s12915-021-01059-4)
Supplement: Supplementary file 1 — Additional file 1: Figure S1. Graphical overview of the experimental design of our study. Deer mice from high- (H) and low- (L) altitude populations were crossed in captivity to produce F1 interpopulation hybrids that were then mated with siblings to produce the F2 interpopulation hybrids that were used in our experiments before and after a 6-wk acclimation to hypobaric hypoxia (12 kPa O2). These hybrids were grouped based on the altitudinal origin of their α- and β- globin genotype. [file 12915_2021_1059_MOESM1_ESM.pdf]

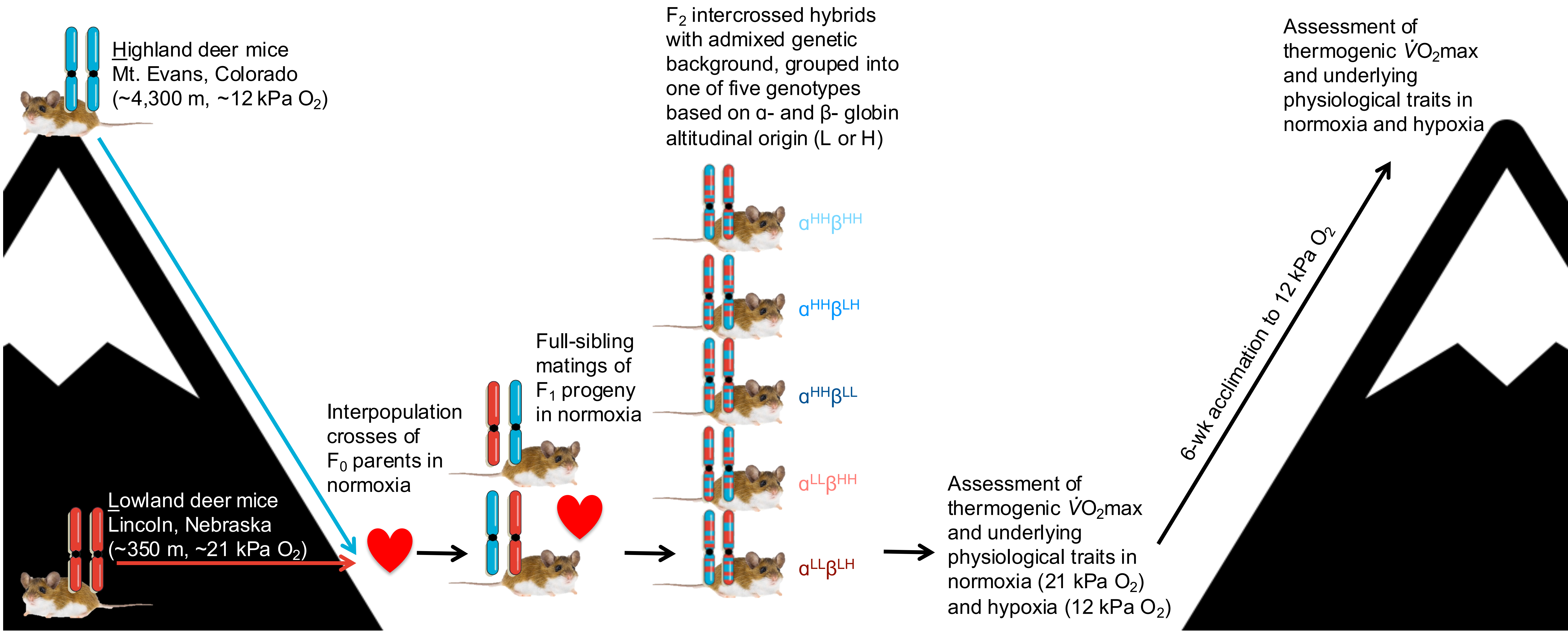

Highland deer mice
Mt. Evans, Colorado
(~4,300 m, ~12 kPa O_2)
Lowland deer mice
Lincoln, Nebraska
(~350 m, ~21 kPa O_2)
Interpopulation crosses of F_0 parents in normoxia
Full-sibling matings of F_1 progeny in normoxia
F_2 intercrossed hybrids with admixed genetic background, grouped into one of five genotypes based on α- and β- globin altitudinal origin (L or H)
$\alpha^{HH}\beta^{HH}$
$\alpha^{HH}\beta^{LH}$
$\alpha^{HH}\beta^{LL}$
$\alpha^{LL}\beta^{HH}$
$\alpha^{LL}\beta^{LH}$
Assessment of thermogenic $\dot{V}O_2$max and underlying physiological traits in normoxia (21 kPa O_2) and hypoxia (12 kPa O_2)
6-wk acclimation to 12 kPa O_2
Assessment of thermogenic $\dot{V}O_2$max and underlying physiological traits in normoxia and hypoxia
