# Supplementary figures and images for: The adaptive benefit of evolved increases in hemoglobin-O2 affinity is contingent on tissue O2 diffusing capacity in high-altitude deer mice
Source: BMC Biol. 2021 Jun 22;19:128. doi: 10.1186/s12915-021-01059-4 (PMC8218429; doi:10.1186/s12915-021-01059-4)

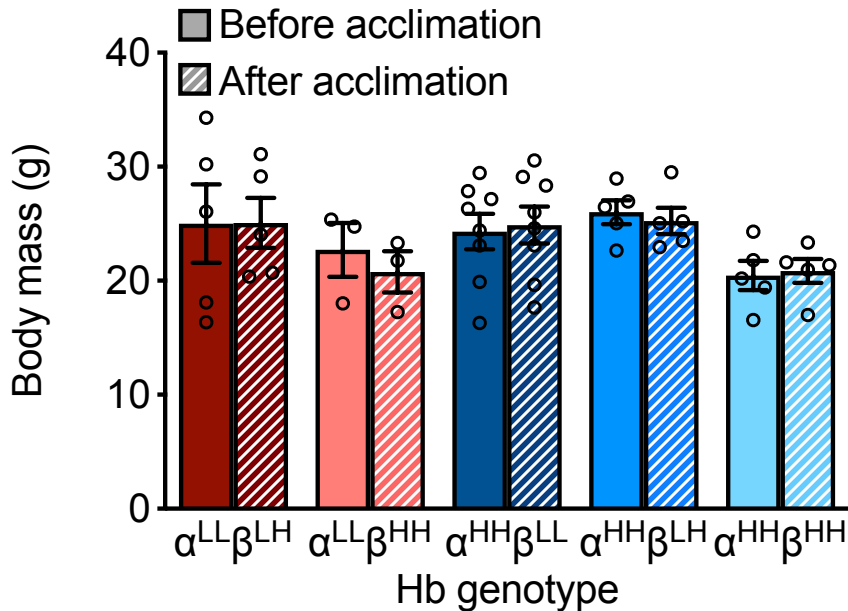

Supplement: Supplementary file 2 — Additional file 2: Figure S2. Body mass of F2 inter-population hybrids both before and after a 6-wk acclimation to hypobaric hypoxia. Each individual’s mass was measured before normoxic and hypoxic V̇O2max trials, with the mean of these values used to create each individual’s data point in the figure. Different α- and β- globin genotypes are shown as superscripts with ‘L’ representing the lowland haplotype and ‘H’ representing the highland haplotype. There was no effect of genotype (P = 0.2977), acclimation (P = 0.4018), or their interaction (P = 0.3362) on body mass. Bars display mean ± SEM (n = 3-8) with individual data superimposed (circles). [file 12915_2021_1059_MOESM2_ESM.pdf]

**A**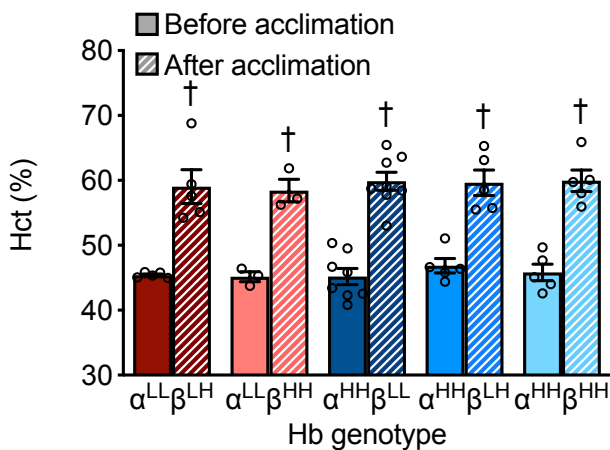**B**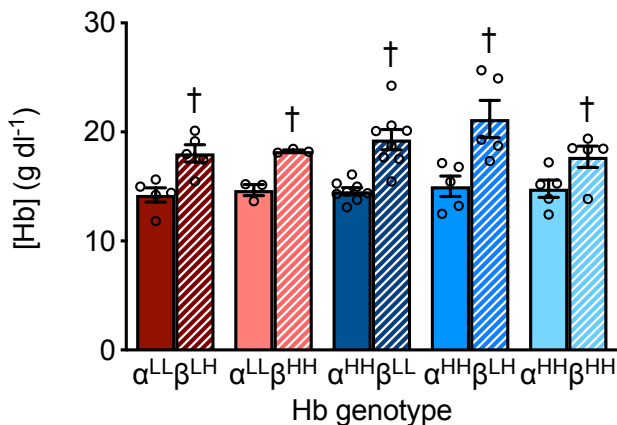**C**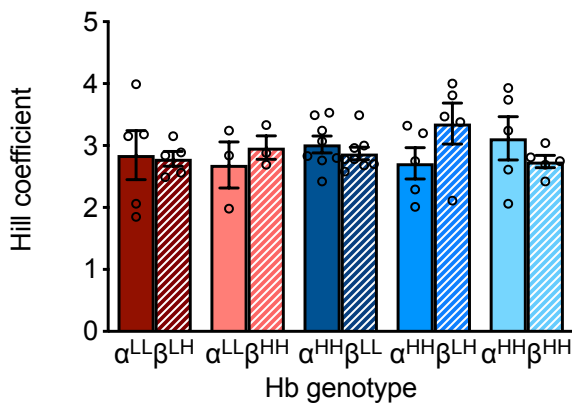

Supplement: Supplementary file 4 — Additional file 4: Figure S3. Hematology of F2 inter-population hybrids measured before and after a 6-wk acclimation to hypobaric hypoxia (12 kPa O2). Hct, hematocrit; [Hb], blood hemoglobin content. Different α- and β- globin genotypes are shown as superscripts with ‘L’ representing the lowland haplotype and ‘H’ representing the highland haplotype. †P < 0.05 vs. pre-acclimation value within a genotype. Bars display mean ± SEM (n = 3-8) with individual data superimposed (circles). [file 12915_2021_1059_MOESM4_ESM.pdf]

**A**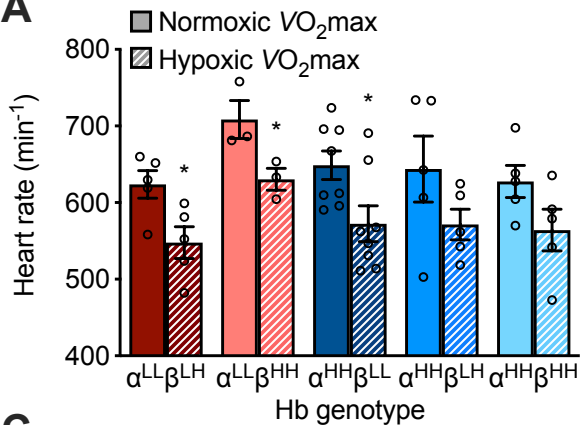**B**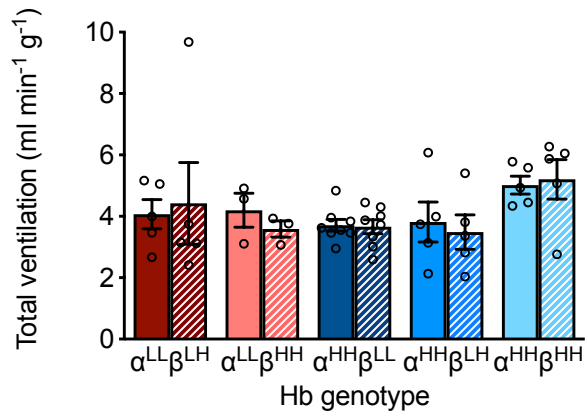**C**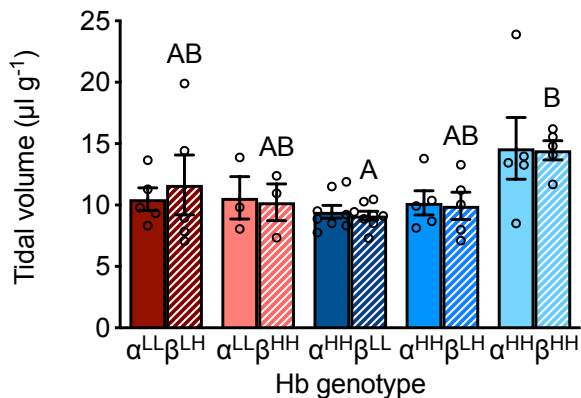**D**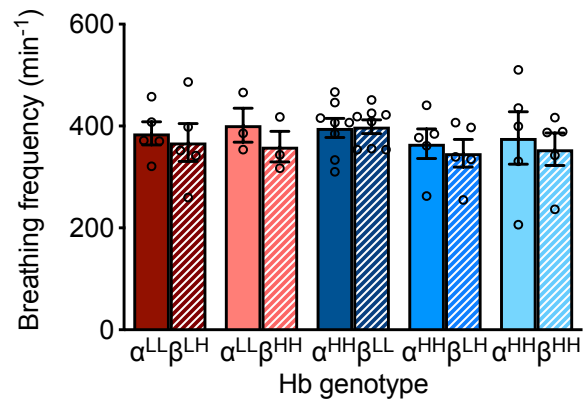

Supplement: Supplementary file 5 — Additional file 5: Figure S4. Physiological parameters for F2 inter-population hybrids acclimated to normoxia, measured at V̇O2max in normoxia (21 kPa O2) and hypoxia (12 kPa O2). Different α- and β- globin genotypes are shown as superscripts with ‘L’ representing the lowland haplotype and ‘H’ representing the highland haplotype. *P < 0.05 vs. normoxia value within a genotype. P < 0.05 between genotypes for hypoxic values not sharing a letter. Bars display mean ± SEM (n = 3-8) with individual data superimposed (circles). [file 12915_2021_1059_MOESM5_ESM.pdf]

$\text{VO}_2\text{max}$

in 12 kPa  $\text{O}_2$  ( $\text{ml min}^{-1} \text{g}^{-1}$ )

■ Before acclimation

▨ After acclimation

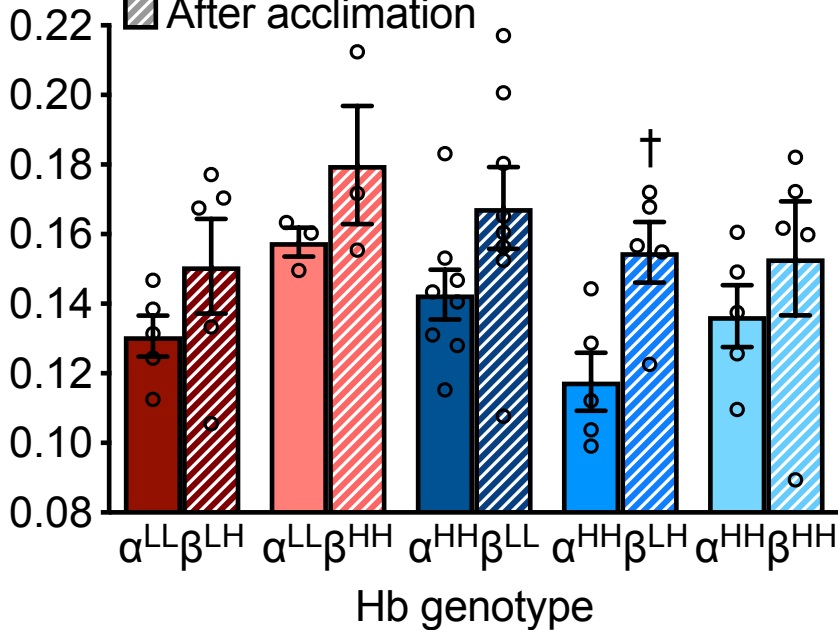

Supplement: Supplementary file 6 — Additional file 6: Figure S5. Hypoxic V̇O2max before and after a 6-wk acclimation to hypobaric hypoxia (12 kPa O2). Different α- and β- globin genotypes are shown as superscripts with ‘L’ representing the lowland haplotype and ‘H’ representing the highland haplotype. †P < 0.05 vs. pre-acclimation value within a genotype. Bars display mean ± SEM (n = 3-8) with individual data superimposed (circles). [file 12915_2021_1059_MOESM6_ESM.pdf]

**A**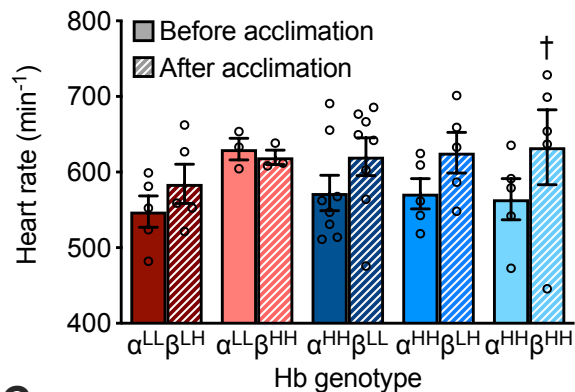**B**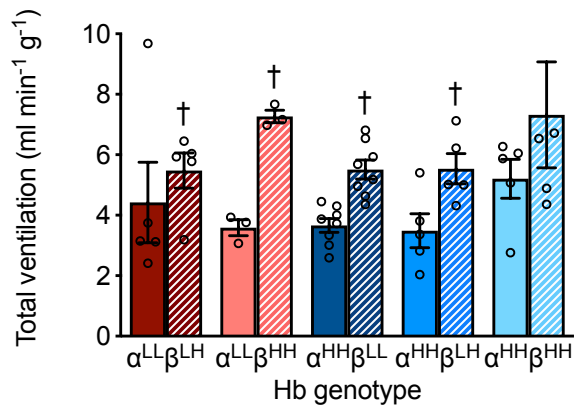**C**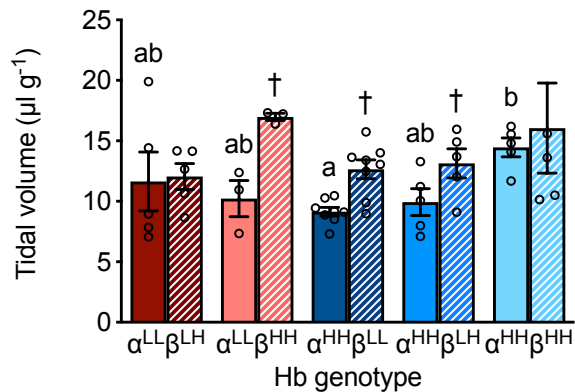**D**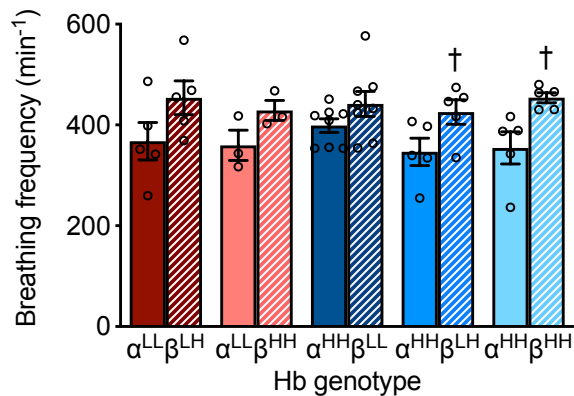

Supplement: Supplementary file 7 — Additional file 7: Figure S6. Physiological parameters for F2 inter-population hybrids measured at V̇O2max in hypoxia (12 kPa O2) both before and after a 6-wk acclimation to hypobaric hypoxia. Different α- and β- globin genotypes are shown as superscripts with ‘L’ representing the lowland haplotype and ‘H’ representing the highland haplotype. †P < 0.05 vs. pre-acclimation value within a genotype. P < 0.05 between genotypes within an acclimation condition for values not sharing a letter. Bars display mean ± SEM (n = 3-8) with individual data superimposed (circles). [file 12915_2021_1059_MOESM7_ESM.pdf]

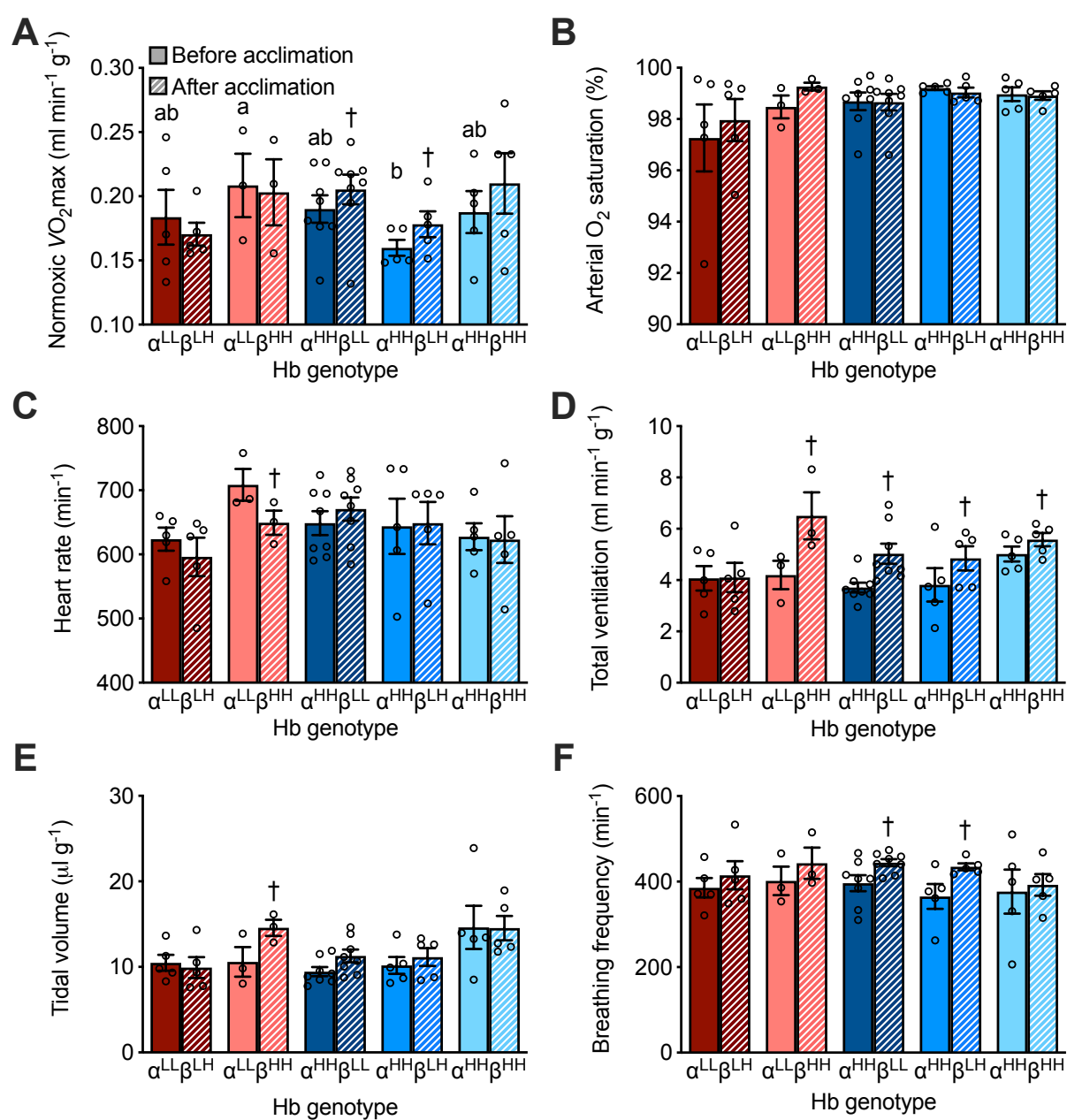

Supplement: Supplementary file 8 — Additional file 8: Figure S7. Physiological parameters for F2 inter-population hybrids measured at V̇O2max in normoxia (21 kPa O2) both before and after a 6-wk acclimation to hypobaric hypoxia. Different α- and β- globin genotypes are shown as superscripts with ‘L’ representing the lowland haplotype and ‘H’ representing the highland haplotype. †P < 0.05 vs. pre-acclimation value within a genotype. P < 0.05 between genotypes within an acclimation condition for values not sharing a letter. Bars display mean ± SEM (n = 3-8) with individual data superimposed (circles). [file 12915_2021_1059_MOESM8_ESM.pdf]
