# Supplementary material for: The adaptive benefit of evolved increases in hemoglobin-O2 affinity is contingent on tissue O2 diffusing capacity in high-altitude deer mice
Source: BMC Biol. 2021 Jun 22;19:128. doi: 10.1186/s12915-021-01059-4 (PMC8218429; doi:10.1186/s12915-021-01059-4)
Supplement: Supplementary file 3 — Additional file 3: Table S1. Effects of inspired PO2 and acclimation to hypoxia on cardiorespiratory physiology of F2 inter-population hybrid deer mice at V̇O2max, without accounting for effects of genotype. Table S2. Effects of acclimation to hypoxia and globin genotype on cardiorespiratory physiology in hypoxia of F2 inter-population hybrid deer mice. Table S3. Effects of inspired PO2 and hemoglobin genotype on cardiorespiratory physiology of F2 inter-population hybrid deer mice acclimated to normoxia. Table S4. Effects of acclimation to hypoxia and globin genotype on cardiorespiratory physiology in normoxia of F2 inter-population hybrid deer mice. Table S5. Parameters used to generate the initial solution in the model of the oxygen transport pathway representing the "ancestral" condition with the most lowland P50. [file 12915_2021_1059_MOESM3_ESM.pdf]

Supplementary Tables for

The adaptive benefit of evolved increases in hemoglobin-O_2 affinity is contingent on tissue O_2 diffusing capacity in high-altitude deer mice

Oliver H. Wearing, Catherine M. Ivy, Natalia Gutiérrez-Pinto, Jonathan P. Velotta, Shane C. Campbell-Staton, Chandrasekhar Natarajan, Zachary A. Cheviron, Jay F. Storz, and Graham R. Scott

Corresponding author: Oliver H. Wearing

Email: wearingo@mcmaster.ca

Table S1. Effects of inspired PO_2 and acclimation to hypoxia on cardiorespiratory physiology of F_2 inter-population hybrid deer mice at $\dot{V}O_2max$, without accounting for effects of genotype.

Trait	Animal mass	Acclimation	PO_2	Interaction[1]
$\dot{V}O_2max$[2]	$F_{1,98} = 37.9184$<br>$P < 0.0001$*	$F_{1,98} = 16.8561$<br>$P = 0.0001$*	$F_{1,98} = 94.4737$<br>$P < 0.0001$*	NS
Arterial O_2 saturation	$F_{1,98} = 3.0602$<br>$P = 0.0909$	$F_{1,98} = 14.5282$<br>$P = 0.0003$*	$F_{1,98} = 181.5338$<br>$P < 0.0001$*	$F_{1,98} = 13.0275$<br>$P = 0.0006$*
Heart rate[2]	$F_{1,97} = 4.3086$<br>$P = 0.0457$*	$F_{1,97} = 4.6517$<br>$P = 0.0342$*	$F_{1,97} = 27.5056$<br>$P < 0.0001$*	$F_{1,97} = 7.6175$<br>$P = 0.0073$*
Total ventilation	NS	$F_{1,99} = 49.9311$<br>$P < 0.0001$*	$F_{1,99} = 3.5126$<br>$P = 0.0648$	$F_{1,99} = 5.5837$<br>$P = 0.0207$*
Tidal volume	NS	$F_{1,99} = 22.2705$<br>$P < 0.0001$*	$F_{1,99} = 3.3406$<br>$P = 0.0716$	$F_{1,99} = 5.1393$<br>$P = 0.0263$*
Breathing frequency	NS	$F_{1,100} = 24.9573$<br>$P < 0.0001$*	$F_{1,100} < 0.0001$<br>$P = 0.9936$	NS

*P < 0.05. [1]Interaction between acclimation and inspired PO_2. NS denotes no significant effect of the factor, which was removed from the final statistical model. [2]Mouse family was also included as a random factor in the mixed model as 0.05 < P < 0.1.

Table S2. Effects of acclimation to hypoxia and globin genotype on cardiorespiratory physiology in hypoxia of F_2 inter-population hybrid deer mice.

Trait	Animal mass	Acclimation	Hb genotype	Interaction[1]
Hypoxic $\dot{V}O_2max$	$F_{1,41} = 28.0073$<br>$P < 0.0001$*	$F_{1,41} = 13.0967$<br>$P = 0.0013$*	$F_{4,41} = 1.7576$<br>$P = 0.1764$	NS
Arterial O_2 saturation	NS	$F_{1,42} = 15.8951$<br>$P = 0.0005$*	$F_{4,42} = 3.0259$<br>$P = 0.0407$*	NS
Heart rate[2]	NS	$F_{1,41} = 10.7106$<br>$P = 0.0031$*	$F_{4,41} = 0.7020$<br>$P = 0.5999$	NS
Total ventilation	NS	$F_{1,42} = 34.0654$<br>$P < 0.0001$*	$F_{4,42} = 0.3396$<br>$P = 0.8481$	NS
Tidal volume	NS	$F_{1,42} = 15.9684$<br>$P = 0.0005$*	$F_{4,42} = 0.6548$<br>$P = 0.6300$	NS
Breathing frequency	NS	$F_{1,42} = 23.7780$<br>$P < 0.0001$*	$F_{4,42} = 0.5053$<br>$P = 0.7323$	NS
Hematocrit	NS	$F_{1,42} = 222.7637$<br>$P < 0.0001$*	$F_{4,42} = 0.2031$<br>$P = 0.9338$	NS
Blood Hb concentration	NS	$F_{1,42} = 59.4601$<br>$P < 0.0001$*	$F_{4,42} = 1.3807$<br>$P = 0.2745$	NS
Red blood cell P_{50}	NS	$F_{1,42} = 18.8344$<br>$P = 0.0002$*	$F_{4,42} = 3.9622$<br>$P = 0.0150$*	NS
Hill coefficient	NS	$F_{1,42} = 0.0315$<br>$P = 0.8599$	$F_{4,42} = 0.2514$<br>$P = 0.9073$	NS

*$P < 0.05$. [1]Interaction between acclimation and globin genotype. NS denotes no significant effect of the factor, which was removed from the final statistical model. [2]Mouse family was also included as a random factor in the mixed model, for which $0.05 < P < 0.1$.

Table S3. Effects of inspired PO_2 and hemoglobin genotype on cardiorespiratory physiology of F_2 inter-population hybrid deer mice acclimated to normoxia.

Trait	Animal mass	PO_2	Hb genotype	Interaction[1]
$\dot{V}O_2max$	$F_{1,41}$ = 31.2238<br>P < 0.0001*	$F_{1,41}$ = 61.0119<br>P < 0.0001*	$F_{4,41}$ = 3.0346<br>P = 0.0416*	NS
Arterial O_2 saturation	NS	$F_{1,41}$ = 122.1012<br>P < 0.0001*	$F_{4,41}$ = 3.7422<br>P = 0.0189*	$F_{4,41}$ = 3.0674<br>P = 0.0389*
Heart rate[2]	NS	$F_{1,41}$ = 32.4464<br>P < 0.0001*	$F_{4,41}$ = 2.8000<br>P = 0.0545	NS
Total ventilation	NS	$F_{1,42}$ = 0.3248<br>P = 0.5738	$F_{4,42}$ = 0.6799<br>P = 0.6136	NS
Tidal volume	NS	$F_{1,42}$ = 0.1428<br>P = 0.7087	$F_{4,42}$ = 1.4425<br>P = 0.2551	NS
Breathing frequency	$F_{1,41}$ = 5.9183<br>P = 0.0242*	$F_{1,41}$ = 0.8443<br>P = 0.3669	$F_{4,41}$ = 1.0643<br>P = 0.3999	NS
Hematocrit	NS	NA	$F_{4,21}$ = 0.3604<br>P = 0.8339	NA
Blood Hb concentration	NS	NA	$F_{4,21}$ = 0.2008<br>P = 0.9351	NA
Red blood cell P_{50}	NS	NA	$F_{4,21}$ = 5.1298<br>P = 0.0048*	NA
Hill coefficient	NS	NA	$F_{4,21}$ = 0.4016<br>P = 0.8053	NA

*P < 0.05. [1]Interaction between inspired PO_2 and globin genotype. NS denotes no significant effect of the factor, which was removed from the final statistical model. NA, not applicable.

[2]Mouse family was also included as a random factor in the mixed model, for which P < 0.05.

Table S4. Effects of acclimation to hypoxia and globin genotype on cardiorespiratory physiology in normoxia of F_2 inter-population hybrid deer mice.

Trait	Animal mass	Acclimation	Hb genotype	Interaction[1]
Normoxic $\dot{V}O_2max$[2]	$F_{1,40} = 23.2664$<br>$P < 0.0001$*	$F_{1,40} = 4.2325$<br>$P = 0.0502$	$F_{4,40} = 1.6212$<br>$P = 0.2115$	NS
Arterial O_2 saturation	$F_{1,41} = 15.3290$<br>$P = 0.0003$*	$F_{1,41} = 0.4014$<br>$P = 0.5296$	$F_{4,41} = 2.9739$<br>$P = 0.0291$*	NS
Heart rate[2]	$F_{1,40} = 4.8558$<br>$P = 0.0381$*	$F_{1,40} = 0.1977$<br>$P = 0.6605$	$F_{4,40} = 2.6116$<br>$P = 0.0698$	NS
Total ventilation	NS	$F_{1,42} = 22.9660$<br>$P < 0.0001$*	$F_{4,42} = 0.3536$<br>$P = 0.8386$	NS
Tidal volume	$F_{1,41} = 5.1639$<br>$P = 0.0317$*	$F_{1,41} = 3.1282$<br>$P = 0.0892$	$F_{4,41} = 1.5707$<br>$P = 0.2199$	NS
Breathing frequency	NS	$F_{1,42} = 8.0995$<br>$P = 0.0087$*	$F_{4,42} = 0.6049$<br>$P = 0.6634$	NS

*$P < 0.05$. [1]Interaction between acclimation and globin genotype. NS denotes no significant effect of the factor, which was removed from the final statistical model. [2]Mouse family was also included as a random factor in the mixed model as $P < 0.05$.

Table S5. Parameters used to generate the initial solution in the model of the oxygen transport pathway representing the 'ancestral condition' with the most lowland P_{50}.

Variable	Value
Measured input parameters	
P_B (kPa)	101
F_IO_2	0.123
$\dot{V}$ (ml min^{-1} g^{-1})	4.96
V_T (μl g^{-1})	13.0
[Hb] (g dl^{-1})	14.2
P_{50} (kPa)	4.84
n	2.85
T_b (˚C)	31.4
Estimated input parameters	
V_D (μl g^{-1})*	6.40
$\dot{Q}$ (ml min^{-1} g^{-1}) †	1.06
Calculated input parameters	
D_LO_2 (ml kPa^{-1} min^{-1})	0.0661
D_TO_2 (ml kPa^{-1} min^{-1})	0.0322
Output parameters (ancestral values shown)	
**P_AO_2** (kPa)	7.18
**P_aO_2** (kPa) †	6.85
**P_vO_2** (kPa)†	2.29
**$\dot{V}O_2$max** (ml min^{-1} g^{-1})	0.131

P_B, barometric pressure; F_IO_2, inspired oxygen fraction; $\dot{V}O_2$max, maximal oxygen consumption rate measured during acute cold exposure; $\dot{V}$, total ventilation; V_T, tidal volume; [Hb], blood hemoglobin concentration; P_{50}, PO_2 at 50% O_2 saturation; n, Hill coefficient; P_aO_2, arterial O_2 tension; T_b, body temperature; V_D, dead space volume; $\dot{Q}$, cardiac ouput; P_vO_2, mixed venous O_2 tension; P_AO_2, alveolar O_2 tension; D_LO_2, O_2 diffusing capacity of the lungs; D_TO_2, O_2 diffusing capacity of the tissues. *Indicates value was taken from Fallica *et al* 2011 (1). †Indicates value was taken from, or calculated using, data in Tate *et al* 2020 (2). Variables in bold were then calculated by the model in our sensitivity analysis in response to changes in D_TO_2 and/or P_{50}.

References

1. J. Fallica, S. Das, M. Horton, W. Mitzner, Application of carbon monoxide diffusing capacity in the mouse lung. *J. Appl. Physiol.* **110**, 1455-1459 (2011).
2. K. B. Tate *et al.*, Coordinated changes across the O2 transport pathway underlie adaptive increases in thermogenic capacity in high-altitude deer mice. *Proc. R. Soc. London., B, Biol. Sci.* **287**, 20192750 (2020).
